# U11/U12 Small Nuclear Ribonucleoprotein TaU11/U12-35K Interacts with TaHis and Negatively Contributes to Fusarium Head Blight Resistance in Wheat

**DOI:** 10.3390/ijms26178288

**Published:** 2025-08-26

**Authors:** Puwen Song, Ao Li, Jiale Deng, Dan Li, Ping Hu, Yuanyuan Guan, Meng Zhang, Qili Liu, Haiyan Hu, Zhengang Ru

**Affiliations:** 1 School of Agriculture, State Key Laboratory of Wheat-Maize Double Cropping and High-Efficiency Production, Henan Engineering Research Center of Crop Genome Editing, Henan Institute of Science and Technology, Xinxiang 453003, China; spw@hist.edu.cn (P.S.); 18236377668@163.com (A.L.);; 2 Henan International Joint Laboratory of Plant Genetic Improvement and Soil Remediation, Henan Institute of Science and Technology, Xinxiang 453003, China; 3 Center of Wheat Research, Henan Institute of Science and Technology, Xinxiang 450003, China

**Keywords:** Fusarium head blight, TaHis, TaU11/U12-35K

## Abstract

Fusarium head blight (FHB), caused by *Fusarium graminearum (F. graminearum)*, has become one of the most devastating wheat diseases, severely impacting both grain yield and quality. The resistance gene *TaHis* (encoding a histidine-rich calcium-binding protein), located at the major FHB resistance locus *Fhb1*, has been demonstrated to confer FHB resistance in wheat, although its underlying mechanism remains unclear. In this study, we screened a wheat yeast two-hybrid (Y2H) library and identified TaU11/U12-35K, a core component of the U12-type spliceosome (U11/U12 small nuclear ribonucleoprotein), as a novel interacting partner of TaHis. Their physical interaction was further confirmed by both Y2H and bimolecular fluorescence complementation assays. Barley stripe mosaic virus-induced gene silencing (BSMV-VIGS)-mediated knockdown of *TaU11/U12-35K* significantly enhanced FHB resistance in both resistant (Bainong 4299) and susceptible (Bainong 5819) cultivars compared to controls. Expression profiling revealed that *TaU11/U12-35K* was significantly downregulated upon *F. graminearum* infection in both cultivars, with consistently lower basal expression levels in Bainong 4299, suggesting a negative correlation between *TaU11/U12-35K* expression and FHB resistance. Collectively, our results demonstrate that TaU11/U12-35K physically interacts with TaHis and functions as a negative regulator of FHB resistance. This study provides new insights into the molecular mechanism of *TaHis*-mediated FHB resistance in wheat.

## 1. Introduction

Wheat (*Triticum aestivum.* L), a staple crop critical for global food security, is increasingly threatened by *F. graminearum*, the primary causal agent of Fusarium head blight (FHB) [1]. This devastating disease not only reduces grain yield but also contaminates harvests with deoxynivalenol (DON), a mycotoxin harmful to human health [2]. Climate change has exacerbated FHB outbreaks by expanding the pathogen’s suitable habitat, further jeopardizing wheat production worldwide [3,4]. During infection, *F. graminearum* colonizes wheat spikes, secreting DON to disrupt host defenses and accelerate disease spread [5,6]. DON triggers premature cell death, enabling the pathogen to shift from biotrophic to necrotrophic growth, thereby enhancing its virulence [7,8,9]. Understanding the molecular basis of wheat resistance to FHB is therefore essential for developing durable disease control strategies.

Genetic studies have identified *Fhb1* on chromosome 3BS as the most effective and widely deployed quantitative trait locus (QTL) for FHB resistance [10]. The cloning of *Fhb1* revealed it encodes TaHis (a histidine-rich calcium-binding protein, His), though its mode of action remains debated. Competing models have emerged: Li et al. proposed that resistance stems from a gain-of-function mutation caused by a partial deletion in *TaHis^S^* [11], whereas Su et al. attributed resistance to reduced expression of the susceptibility allele (*TaHis^S^*/*TaHRC^S^*) via allelic variation (*TaHis^R^*/*TaHRC^R^*) [12]. This mechanistic divergence highlights the complexity of *TaHis*-mediated resistance and suggests potential cultivar-specific resistance pathways.

Recent advances have begun to unravel *TaHis*/*TaHRC*’s role in defense signaling networks. In susceptible wheat, TaHis^S^ interacts with the calcium transporter TaCAXIP4, disrupting Ca^2+^ homeostasis and suppressing the NADPH oxidase-dependent oxidative burst, a critical component of early defense responses [13]. Strikingly, He et al. demonstrated that TaHis^S^/TaHRC^S^ undergoes liquid–liquid phase separation via its intrinsically disordered region (IDR), forming nuclear condensates that sequester splicing factors and alter the expression of defense-related genes (e.g., *PR1*/*PR5*). The resistance allele *TaHis^R^*/*TaHRC^R^*, lacking a functional IDR, fails to form these condensates, thereby maintaining proper immune gene regulation [14]. These findings position *TaHis* as a central regulator at the intersection of calcium signaling and transcriptional reprogramming during pathogen attack.

To further elucidate *TaHis*’s resistance mechanism, we employed yeast two-hybrid (Y2H) screening using TaHis^R^ as bait and identified TaU11/U12-35K, a U11/U12 spliceosome component, as a novel interacting partner. This physical interaction was confirmed by both Y2H and bimolecular fluorescence complementation (BiFC) assays. Functional studies using BSMV-mediated virus-induced gene silencing (VIGS) revealed that *TaU11/U12-35K* knockdown enhances FHB resistance in both resistant and susceptible wheat cultivars, suggesting it acts as a negative regulator of immunity. Intriguingly, *TaU11/U12-35K* expression is significantly downregulated during *F. graminearum* infection, with resistant cultivars exhibiting constitutively lower transcript levels—a pattern reminiscent of *TaHis^S^/TaHRC^S^* suppression in *Fhb1* lines. These findings support a model wherein the TaHis^R^–TaU11/U12-35K interaction modulates spliceosome activity to sustain defense gene expression, providing new insights into the molecular basis of *Fhb1*-mediated resistance.

## 2. Results

### 2.1. Identification of the TaU11/U12-35K Gene

The TaHis CDS (825 bp) was PCR-amplified from Bainong 4299 cDNA with TaHis-F/R, then recombined into pGBKT7 to generate the bait vector pGBKT7-TaHis. Yeast two-hybrid screening of the Bainong 4299 cDNA library identified 40 positive clones. Plasmids were rescued via *E. coli* transformation, sequenced, and subjected to BLASTn/BLASTp (NCBI/Ensembl Plants). Insert cDNA from four clones encoded the same protein. ORF Finder analysis of the inserted cDNA revealed a 1260 bp ORF encoding 419 amino acids, identified as the coding sequence (CDS) for this protein. Alignment with NCBI and Ensembl Plant databases showed 100% amino acid sequence identity with the U11/U12 small nuclear ribonucleoprotein 35 kDa protein-like (TraesCS5D02G304800) in wheat, hence it was named TaU11/U12-35K.

### 2.2. Y2H Validation of TaHis-TaU11/U12-35K

**Figure 1:**
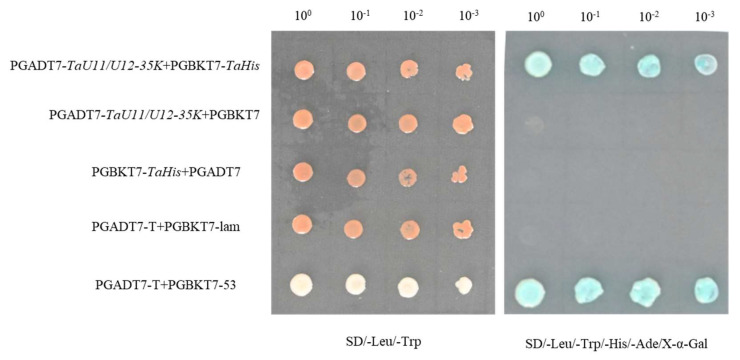
Yeast two-hybrid validation of the interaction between TaHis and TaU11/U12-35K. Shown are the experimental group (pGBKT7-TaHis + pGADT7-TaU11/U12-35K), negative controls (pGBKT7-TaHis + pGADT7, pGADT7-TaU11/U12-35K + pGBKT7, and pGBKT7-Lam + pGADT7-T), and positive control (pGBKT7-53 + pGADT7-T). Yeast transformants were plated on SD/-Trp/-Leu and SD/-Trp/-Leu/-Ade/-His media supplemented with X-α-Gal. Dilution factors (1, 10^−1^, 10^−2^, 10^−3^) are indicated.

The TaU11/U12-35K CDS (1260 bp) was amplified from Bainong 4299 cDNA with AD-TaU11/U12-35K-F/R, then cloned into pGADT7 via homologous recombination to generate pGADT7-TaU11/U12-35K. For Y2H assays, we co-transformed Y2HGold yeast cells with the following plasmid combinations: experimental group (pGBKT7-TaHis + pGADT7-TaU11/U12-35K), negative controls (pGBKT7-TaHis + pGADT7, pGADT7-TaU11/U12-35K + pGBKT7, and pGBKT7-Lam + pGADT7-T), and positive control (pGBKT7-53 + pGADT7-T). Transformants were cultured at 30 °C for 3–5 days on selection media. Both the experimental group and positive control exhibited robust growth on double dropout medium (SD/-Leu/-Trp) and developed blue coloration on quadruple dropout medium (SD/-Leu/-Trp/-His/-Ade/X-α-Gal). In contrast, while all negative controls grew on double dropout medium, none survived on the quadruple dropout medium (Figure 1). These results demonstrate a specific protein–protein interaction between TaHis and TaU11/U12-35K in the yeast system.

### 2.3. BiFC Validation of TaHis-TaU11/U12-35K Interaction

**Figure 2:**
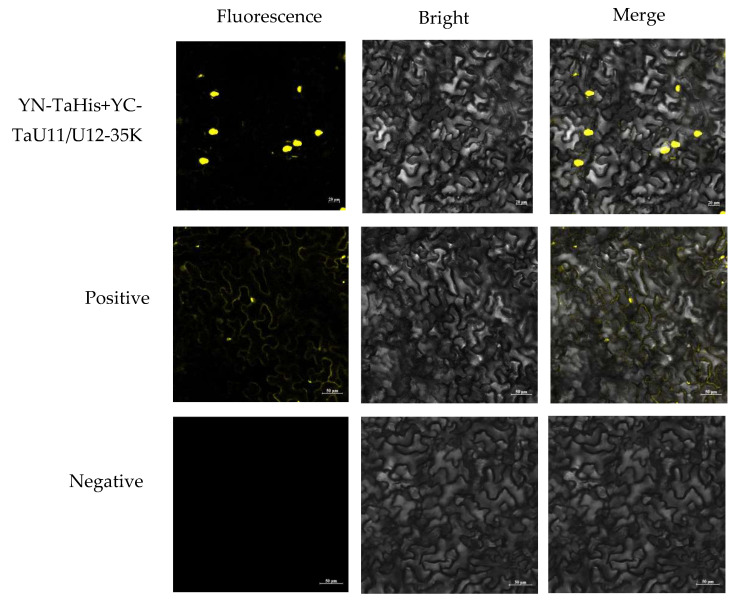
Bimolecular fluorescence complementation (BiFC) analysis of the TaU11/U12-35K and His interaction in *N. benthamiana* epidermal cells. Shown are the experimental group (YN-TaHis + YC-TaU11/U12-35K), positive control (YN-TaPFT + YC-TaRBL), and negative control (YN-TaHis + YC). Representative images display YFP fluorescence in single confocal sections (**left**), corresponding transmission light images (**middle**), and merged views (**right**). The fluorescent signal is yellow (YFP), and the cellular structure is gray (transmission light).

TaHis and TaU11/U12-35K CDSs were amplified with BiFC-specific primers (TaHis-F/R; TaU11/U12-35K-F/R) and Gateway-cloned into YN/YC vectors to generate fusion constructs YN-TaHis and YC-TaU11/U12-35K. The recombinant vectors were transformed into *Agrobacterium tumefaciens* strain GV3101 and subsequently co-infiltrated into *Nicotiana benthamiana* leaves using the following experimental design: experimental group (YN-TaHis + YC-TaU11/U12-35K), positive control (YN-TaPFT + YC-TaRBL), and negative control (YN-TaHis + YC). Following 72 h of incubation, we observed distinct yellow fluorescent signals in both the experimental group and positive control under confocal microscopy. In contrast, no fluorescence was detected in the negative control. These results provide compelling in planta evidence for a specific interaction between TaU11/U12-35K and TaHis proteins in living plant cells (Figure 2).

### 2.4. Silencing of TaU11/U12-35K Enhances Wheat FHB Resistance

**Figure 3:**
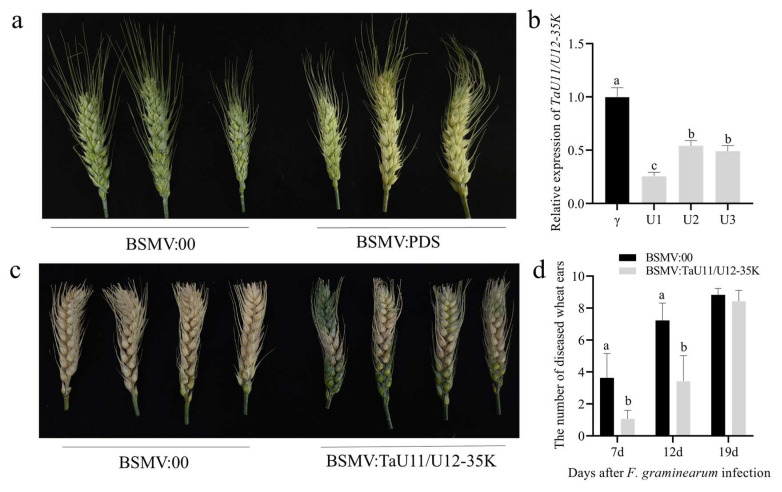
Effects of *TaU11/U12-35K* silencing (VIGS) in wheat cultivar Bainong 5819 and FHB assessment: (**a**) Spikelet phenotypes at 15 days post-inoculation (dpi) with BSMV:00 (control) and BSMV: PDS constructs. (**b**) Relative *TaU11/U12-35K* expression in control and silenced plants at 19 dpi (pre-pathogen inoculation for VIGS groups). (**c**) Disease symptoms on control (**left**) and *TaU11/U12-35K*-silenced (**right**) spikes at 19 dpi. (**d**) Disease progression quantified by number of diseased panicles in *TaU11/U12-35K*-silenced plants at 7, 12, and 19 dpi. Error bars represent the standard deviation from three replicated experiments. Lowercase letters indicate significant differences between treatments within the same variety (*p* < 0.05; ANOVA).

**Figure 4:**
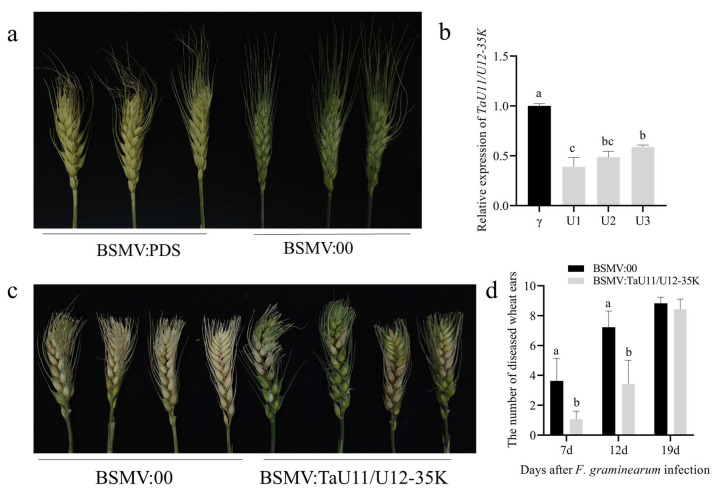
Effects of *TaU11/U12-35K* silencing (VIGS) in wheat cultivar Bainong 4299 and FHB assessment: (**a**) Spikelet phenotypes at 15 days post-inoculation (dpi) with BSMV:00 (control) and BSMV: PDS constructs. (**b**) Relative *TaU11/U12-35K* expression in control and silenced plants at 19 dpi (pre-pathogen inoculation for VIGS groups). (**c**) Disease symptoms on control (**left**) and *TaU11/U12-35K*-silenced (**right**) spikes at 23 dpi. (**d**) Disease progression quantified by number of diseased panicles in *TaU11/U12-35K*-silenced plants at 7, 12, and 19 dpi. Error bars represent the standard deviation from three replicated experiments. Lower case letters indicate significant differences between treatments within the same variety (*p* < 0.05; ANOVA).

To investigate the role of TaU11/U12-35K in FHB resistance, we employed the BSMV-VIGS system in both the FHB-resistant cultivar Bainong 4299 and susceptible cultivar Bainong 5819. The experimental design included: a *TaU11/U12-35K* experimental group BSMV: TaU11/U12-35K (α+β+γ-*TaU11/U12-35K*), a negative control group BSMV:00 (α+β+γ), and a positive control group BSMV: PDS (α+β+γ-*PDS*). Fifteen days after silencing, the positive control exhibited the typical photo-bleaching phenotype (Figure 3a and Figure 4a), confirming successful VIGS system operation. At anthesis, we challenged the TaU11/U12-35K-silenced and control plants with *F. graminearum* conidial suspensions. Quantitative analysis revealed that *TaU11/U12-35K* silencing achieved approximately 50% reduction in transcript levels in both cultivars (Figure 3b and Figure 4b). Notably, the silencing of *TaU11/U12-35K* significantly enhanced FHB resistance in both genotypes, with more pronounced effects observed in Bainong 4299 (Figure 3c and Figure 4c). The quantification of infected spikelets at 7, 12, and 19 dpi in both cultivars corroborated the aforementioned results (Figure 3d and Figure 4d). Collectively, our molecular and phenotypic evidence strongly suggests that *TaU11/U12-35K* likely functions as a susceptibility factor in wheat-FHB interactions.

### 2.5. Differential Expression of TaU11/U12-35K Gene in Resistant and Susceptible Wheat

**Figure 5:**
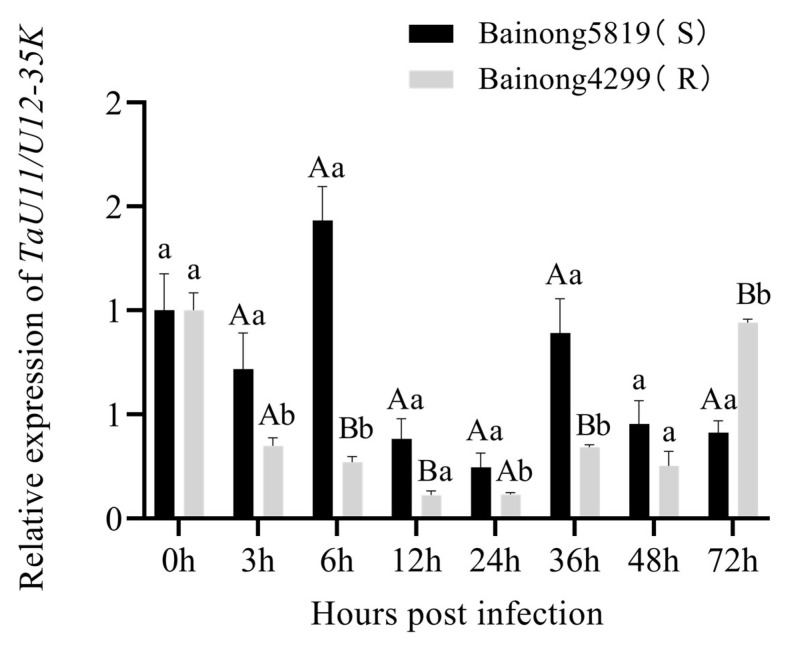
Temporal expression profile of *TaU11/U12-35K* in Bainong4299 and Bainong5819 cultivars following *F. graminearum* inoculation. Relative transcript levels (normalized to *Actin*) were measured at 0, 3, 6, 12, 24, 36, 48, and 72 hpi. Error bars represent standard deviation from three biological replicates. Lowercase letters (a,b) indicate significant differences between cultivars at each time point (*p* < 0.05); uppercase letters (A,B) denote highly significant differences (*p* < 0.01) based on pairwise Student’s *t*-tests. (*p*-values were adjusted for multiple comparisons using the Bonferroni method.).

To characterize the FHB-responsive expression pattern of *TaU11/U12-35K*, we conducted time-course analyses in spikelets of Bainong 4299 and Bainong 5819 wheat cultivars following inoculation with *F. graminearum*. Tissue samples were collected at 0, 3, 6, 12, 24, 36, 48, and 72 hpi for RT-qPCR analysis. The results revealed distinct cultivar-specific expression dynamics (Figure 5): in Bainong 4299, rapid downregulation was observed, with maximal suppression (89% reduction) at 12 hpi, and gradual recovery to near-baseline levels occurred by 72 hpi. Whereas, in Bainong 5819, transient upregulation occurred during early infection (3–6 hpi), subsequent decline reached maximum suppression (75% reduction) at 24 hpi. Comparative analysis showed that, except at 0 h, the expression level of *TaU11/U12-35K* at the same time points was significantly higher in Bainong 5819 than in Bainong 4299. However, there was a striking reversal at 72 hpi, where Bainong 4299 showed 2.24-fold higher expression than Bainong 5819. These temporal expression patterns suggest *TaU11/U12-35K* may participate in early susceptibility mechanisms in the vulnerable cultivar and late-stage defense responses in the resistant genotype.

## 3. Discussion

Alternative splicing (AS), a crucial post-transcriptional regulatory mechanism in plants, significantly enhances proteomic diversity and adaptive responses to environmental challenges [15,16,17]. Through mechanisms including exon skipping (ES) and intron retention (IR), plants generate distinct splice isoforms to fine-tune their defense responses against diverse biotic stresses, including bacterial, fungal, and viral pathogens [18]. Emerging evidence reveals AS plays a dual role in plant immunity, as exemplified by the potato late blight resistance gene *RB* which undergoes infection-induced AS reprogramming to produce full-length resistance proteins, while pathogen effectors like *Phytophthora sojae*’s PsAvr3c and *P. infestans*’ SRE3 manipulate host AS machinery to promote infection [19,20,21]. In wheat–*F. graminearum* interactions, a novel AS-mediated susceptibility mechanism where the susceptibility allele *TaHis*-S promotes liquid–liquid phase separation (LLPS), enhancing interactions with splicing factor TaSR45a and recruiting excessive splice factors into nuclear condensates that increase aberrant AS events (IR/ES) and disrupt defense-related transcriptomes. Conversely, the resistance allele *TaHis*-R suppresses LLPS to maintain splice factor availability and canonical splicing patterns of defense genes, with *TaHis*-R-mediated AS fidelity correlating with stable defense gene expression and FHB resistance [14]. These findings collectively establish that while plants employ AS to enhance immunity, pathogens like *F. graminearum* can subvert this mechanism, and maintaining spliceosome stability emerges as a critical determinant of disease outcomes and potential breeding target for FHB resistance.

To further elucidate the molecular mechanism of the FHB resistance gene *TaHis*-R, we employed it as bait to screen a wheat Y2H cDNA library, which led to the identification of its interaction with the small nuclear ribonucleoprotein (snRNP) TaU11/U12-35K. This protein–protein interaction was subsequently confirmed by BiFC assay. U11/U12-35K represents a core component of the U12-dependent spliceosome in plants and plays a crucial role in alternative splicing, particularly in the processing of U12-type introns [22]. Studies have demonstrated that *U11/U12-35K* facilitates 5’ splice site selection during U12-type intron splicing through its interaction with SR proteins, exhibiting functional analogy to U1-70K in U2-type intron splicing [22]. Functional characterization via VIGS demonstrated that knockdown of *TaU11/U12-35K* in both Bainong 4299 and Bainong 5819 cultivars significantly enhanced FHB resistance while reducing target gene expression, suggesting *TaU11/U12-35K* acts as a susceptibility factor that negatively regulates host immunity. Temporal expression profiling revealed that *TaU11/U12-35K* transcript levels were substantially downregulated following *F. graminearum* infection in both cultivars, with consistently higher basal expression observed in the susceptible genotype throughout most infection time points. This inverse correlation between *TaU11/U12-35K* expression and FHB resistance suggests that during pathogen infection, *F. graminearum* may hijack TaU11/U12-35K to reprogram host pre-mRNA splicing by increasing aberrant splicing events (e.g., intron retention and exon skipping), thereby promoting susceptibility.

Our findings collectively demonstrate that TaHis-R likely coordinates immune-metabolic balance by directly suppressing TaU11/U12-35K activity, representing a novel regulatory mechanism underlying wheat FHB resistance through modulation of spliceosomal function. This study provides important insights into how resistance proteins can counteract pathogen manipulation of host splicing machinery to maintain disease resistance.

## 4. Materials and Methods

### 4.1. Plant and Fungal Materials

FHB-resistant wheat cultivar Bainong 4299 (*Fhb1+*) and FHB-susceptible cultivar Bainong 5819 (*Fhb1*-) were bred and maintained by Henan Institute of Science and Technology. *N. benthamiana* plants and the *F. graminearum* strain were preserved by the Henan Engineering Research Center for Genome Editing of Major Food Crops. Bainong 4299 and Bainong 5819 were used for gene amplification, gene expression, and VIGS studies. Wheat seeds were placed on moist filter paper in Petri dishes and incubated at 21 °C for 96 h for germination. Germinated seeds were transplanted into pots containing seedling substrate and grown under artificial greenhouse conditions: 18–25 °C, 16 h light/8 h dark photoperiod. Wild-type *N. benthamiana* plants were also grown in an artificial greenhouse under conditions set to: 20–24 °C, 14 h light/10 h dark photoperiod. The *F. graminearum* isolate used in this study was a field isolate originating from Henan Province, China. Following Buerstmayr [23], *F. graminearum* was inoculated onto potato dextrose agar (PDA) solid medium and cultured at 25 °C for 4 days, then transferred to mung bean liquid medium for spore induction.

### 4.2. Wheat RNA Extraction, cDNA Synthesis, and Gene Amplification

Total RNA was extracted from all wheat spike samples using the Trizol method, followed by further purification with an RNase-Free DNase I kit. Purified RNA was reverse-transcribed into cDNA using the PrimeScript™ II 1st Strand cDNA Synthesis Kit (Takara, Dalian, China). Specific primers TaHis-F/R (Table 1) were designed based on the *TaHis* gene sequence reported by Li [11] to amplify TaHis using 2×Es Taq MasterMix enzyme. Primer sequences used in this study are listed in Table 1. Sequencing and primer synthesis were performed by Genecreate Biotech (Wuhan, China).

### 4.3. Yeast Two-Hybrid (Y2H) Interaction Validation

The full-length TaHis sequence was amplified by PCR using primers listed in Table 1. The fragment was inserted into pGBKT7 to generate the bait vector pGBKT7-TaHis. After testing for autoactivation and toxicity in yeast, the Matchmaker™ Gold Yeast Two-Hybrid System was used to screen the wheat cDNA library with pGBKT7-TaHis, identifying the interacting protein TaU11/U12-35K. The full-length coding region of TaU11/U12-35K was PCR-amplified using specific primers (Table 1) based on Bainong 4299 cDNA and inserted into the pGADT7 vector to generate pGADT7-TaU11/U12-35K. The recombinant vectors were co-transformed into Y2HGold yeast cells as follows: experimental group (pGBKT7-TaHis + pGADT7-TaU11/U12-35K), negative control groups (pGBKT7-TaHis + pGADT7, pGBKT + pGADT7-TaU11/U12-35K, and pGBKT7 + pGADT7), and positive control group (pGBKT7-53 + pGADT7-T). Positive transformants were selected on SD/-Leu/-Trp medium at 30 °C for 2–3 days. Subsequently, protein interaction was tested on SD/-Leu/-Trp/-His/-Ade medium containing X-α-Gal as a substrate, incubated at 30 °C for 4–5 days.

### 4.4. Bimolecular Fluorescence Complementation (BiFC) Assay

The BiFC assay was performed as previously described by Song et al. [24]. The coding sequences (CDSs) of *TaHis* and *TaU11/U12-35K* were amplified by PCR (Table 1) and inserted into the BiFC vectors pEarleyGate202-nYFP (containing the N-terminal fragment of YFP, YN) to generate *TaHis*-YN and pEarleyGate201-cYFP (containing the C-terminal fragment of YFP, YC) to generate *TaU11/U12-35K*-YC. Constructs were transformed into *Agrobacterium tumefaciens* strain GV3101. The experimental group (TaHis-YN + TaU11/U12-35K-YC), positive control, and negative control (TaHis-YN + YC) were co-infiltrated into leaves of 4-week-old *N. benthamiana* plants using agrobacterium-mediated transient expression. Infiltrated plants were incubated at 25 °C for 4 days before observation. Yellow fluorescent signals were observed using a laser scanning confocal microscope (Carl Zeiss AG, Oberkochen, Germany).

### 4.5. Functional Validation of TaU11/U12-35K Gene Using BSMV-VIGS

A silencing fragment of *TaU11/U12-35K* was amplified using primers BSMV-TaU11/U12-35K-F/R (Table 1). The γ vector was cut with *Sma* I and then joined to the fragment using recombination to make γ-*TaU11/U12-35K*. The α, γ, γ-*PDS*, and γ-*TaU11/U12-35K* vectors were linearized with *Mlu* I, and the β vector was linearized with *Spe* I. The linearized DNA fragments were purified and subjected to in vitro transcription with the mMESSAGE mMACHINE T7 transcription kit (Thermo Fisher Scientific, Waltham, MA, USA). At the wheat booting stage, uniformly growing spikes were selected. A mixture containing 10 μL each of the α, β, and γ-*PDS*/γ-*TaU11/U12-35K* transcripts (1:1:1 ratio), an equal volume of DEPC water, and 225 μL FES buffer was prepared on ice. Under sterile conditions, 15 μL of the mixture was used to inoculate the spikes via the fingertip-rubbing method. Inoculated spikes were sprayed with DEPC water and covered with black plastic bags to maintain humidity for 24 h. Seven days later (at anthesis), spikes were inoculated with *F. graminearum* spore suspension. Disease assessment and spike sampling (stored at −80 °C) were performed at 7, 14, and 19 days post-inoculation (dpi). RNA extraction and cDNA synthesis followed Section 2.2 (cDNA stored at −20 °C). *TaU11/U12-35K* expression was quantified by RT-qPCR using qTaU11/U12-35K-F/R primers and ChamQ Universal SYBR qPCR Master Mix (Vazyme, Nanjing, China), with *Tubulin* as the internal reference gene. The PCR reaction volume was 20 μL, containing the corresponding reagents. Amplification was performed on a QuantStudio 6 Flex system according to the manufacturer’s instructions, with three technical replicates and three biological replicates. Relative gene expression was calculated using the 2^−ΔΔCt^ method.

### 4.6. Analysis of TaU11/U12-35K Gene Expression Pattern Induced by FHB

Following Zhang [25], the *F. graminearum* spore suspension was prepared at a concentration of 1 × 10^5^ conidia/mL. Bainong 4299 and Bainong 5819 wheat cultivars were inoculated at anthesis using the single-floret injection method. Spikelet samples were collected at different time points (0, 3, 6, 12, 24, 36, 48, and 72 h post-inoculation, hpi), with one pair of spikelets sampled per time point (stored at −80 °C). RNA extraction and reverse transcription followed Section 2.2, and the resulting cDNA was aliquoted and stored at −20 °C. RT-qPCR analysis of *TaU11/U12-35K* gene expression was performed using qTaU11/U12-35K-F/R primers (Table 1), the above cDNA as template, and ChamQ Universal SYBR qPCR Master Mix (Vazyme, Nanjing, China), with the *Tubulin* gene as the internal reference. RT-qPCR was conducted following the method described in Section 2.5.

## 5. Conclusions

In this study, we screened a wheat yeast two-hybrid (Y2H) library and identified TaU11/U12-35K, a core component of the U12-type spliceosome (U11/U12 small nuclear ribonucleoprotein), as a novel interacting partner of TaHis. Their physical interaction was further confirmed by both Y2H and bimolecular fluorescence complementation assays. BSMV-VIGS-mediated knockdown of *TaU11/U12-35K* significantly enhanced FHB resistance in both resistant (Bainong 4299) and susceptible (Bainong 5819) cultivars compared to controls. Expression profiling revealed that *TaU11/U12-35K* was significantly downregulated upon *F. graminearum* infection in both cultivars, with consistently lower basal expression levels in Bainong 4299, suggesting a negative correlation between *TaU11/U12-35K* expression and FHB resistance. Collectively, our results demonstrate that TaU11/U12-35K physically interacts with TaHis and functions as a negative regulator of FHB resistance. This study provides new insights into the molecular mechanism of *TaHis*-mediated FHB resistance in wheat.

## Figures and Tables

**Table 1:** Primer sets used in this study.

Primer Name	Primer Sequence (5′—3′)
TaU11/U12-35K-F TaU11/U12-35K-R TaHis-F TaHis-R AD-TaU11/U12-35K-F AD-TaU11/U12-35K-R BD-TaHis-F BD-TaHis-R BIFC-TaU11/U12-35K-F BIFC-TaU11/U12-35K-R BIFC-TaHis-F BIFC-TaHis-R BSMV-TaU11/U12-35K-F BSMV-TaU11/U12-35K-R qTubulin-F qTubulin-R qActin-F qActin-R qTaU11/U12-35K-F qTaU11/U12-35K-R	ATGAGGGTCGGCGTCG CCTCTGCTCCCGATGAG ATCTGTATATTTGGACAACCATGTA TTACACGAGTTGCTTCCCGT GGAGGCCAGTGAATTCATGAGAGTCGGCGTCGGCGGGA CGAGCTCGATGGATCCTTACTGGCTATAATCTCGGCTC CATGGAGGCCGAATTCATGTCTATATTTGGACAACCATGTA GCAGGTCGACGGATCCTTACACGAGTTCCTTCCCGT GGGGACAAGTTTGTACAAAAAAGCAGGCTTC ATGAGGGTCGGCGTCGGCG GGGGACCACTTTGTACAAGAAAGCTGGGTC CCTCTGCTCCCGATGAGAT GGGGACAAGTTTGTACAAAAAAGCAGGCTTCATGTGTATATTTGGACAACCATGTA GGGGACCACTTTGTACAAGAAAGCTGGGTCCACGAGTTGCTTCCCGTC TAGCTGATTAATTAACCCGGG AACTGAAGACGACGCAGGTC TAGCTGAGCGGCCGCCCCGGG TACCTCTGCTCCCGATGAGAT ATCTCCAACTCCACCAGTGTCG TCATCGCCCTCATCACCGTC TGACCGTATGAGCAAGGAG CCAGACAACTCGCAACTTAG CTAGGAGGAGGACTTGGAGGAA CAAAACGTGTCATATACCGCCC

## Data Availability

The original contributions presented in this study are included in the article. Further inquiries can be directed to the corresponding authors.
